# Molecular docking studies of (4*Z*, 12*Z*)-cyclopentadeca-4, 12-dienone from *Grewia hirsuta* with some targets related to type 2 diabetes

**DOI:** 10.1186/s12906-015-0588-5

**Published:** 2015-03-20

**Authors:** Abirami Natarajan, Shobana Sugumar, Sivakumar Bitragunta, Natarajan Balasubramanyan

**Affiliations:** Department of Chemistry, SRM University, Kattankulathur, 603203 India; Department of Bioinformatics, School of Bioengineering, SRM University, Kattankulathur, 603203 India

**Keywords:** Antidiabetic, *Grewia hirsuta*, Docking

## Abstract

**Background:**

Management of diabetes without any side effects is still a challenge to the medical system. This leads to increasing demand for natural products with antidiabetic activity with fewer side effects. *Grewia hirsuta* (Tiliaceae) is a traditional herbal medicinal plant and is reported to possess a variety of pharmacological actions. In the present research, a compound (4Z, 12Z)-cyclopentadeca-4, 12-dienone isolated from *Grewia hirsuta* was taken as ligand for molecular docking studies. Evaluation of hypoglycemic activity through an extensive *in silico* docking approach with molecular targets such as aldose reductase, glucokinase, pyruvate dehydrogenase kinase isoform 2, peroxisome proliferator-activated receptor-gamma, glycogen synthase kinase-3, 11β-Hydroxysteroid dehydrogenase, and glutamine: fructose-6-phosphate amidotransferase were performed.

**Methods:**

Isolation of the (4Z, 12Z)-cyclopentadeca-4,12-dienone from the methanol extract of the leaves of *Grewia hirsuta* was performed by the column chromatography to yield different fractions. These fractions were then subjected to purification and the structure was elucidated and confirmed by spectroscopic methods including UV, FTIR, ^1^H, ^13^C NMR and the accurate mass determination was carried out using the Q-TOF mass spectrometer. *In-vivo* experimentation was performed with evaluation of α-glucosidase, α-amylase and MTT assay that had been reported by the author in the earlier paper. Molecular docking study was performed with GLIDE docking software.

**Results:**

The docking studies of the ligand (4Z, 12Z)-cyclopentadeca-4, 12-dienone with seven different target proteins showed that this is a good inhibitor, which docks well with various targets related to diabetes mellitus. Hence (4Z, 12Z)-cyclopentadeca-4,12-dienone can be considered for developing into a potent anti-diabetic drug.

**Conclusion:**

The results of the current study have revealed that the leaves of the selected plant *Grewia hirsuta* contains a potential inhibitor for diabetes (4*Z*, 12*Z*)-cyclopentadeca-4,12-dienone. Thus enabling a possibility of this plant extract as a new alternative to existing diabetic approaches.

## Background

Diabetes mellitus is a metabolic disorder characterized by a loss of glucose homeostasis with disturbances of carbohydrate, fat and protein metabolism resulting from defects in insulin secretion, insulin action, or both [1]. The word “diabetes” is derived from the Greek word “Diab” (meaning to pass through, referring g to the cycle of heavy thirst and frequent urination); “mellitus” is the Latin word for “sweetened with honey” (refers to the presence of sugar in the urine) [2]. According to ancient Hindu physicians, “Madhumeha” is a disease, in which a patient passes sweet urine and exhibits sweetness all over the body,such as in sweat, mucus, breath, and blood [3]. It was recommended that the low carbohydrate diet and almost total withdrawal of animal fats should be taken by the patients suffering from Madhumeha, whereas obese adults should live on low calorie diet. Diabetes has been known in India for centuries as ‘a disease of rich man’ but is now spread among all masses [4]. It is becoming the third “killer” of the health of mankind a long with cancer, cardiovascular and cerebrovascular diseases [5]. Plants being a natural reservoir of many medicinal value added components help to overcome many chronic disorders. The use of allopathic drugs had caused severe side effects and not found to be cost effective. Hence herbal medicines are considered to be an excellent remedy for diseases like cancer, diabetes, liver diseases and arthritis. The bioactive compounds of medicinal plants are used as anti diabetic, chemotherapeutic, anti inflammatory, anti arthritic agents where no satisfactory cure is present in modern medicines [6]. Herbal drugs are prescribed due to their good effectiveness, fewer side effects in clinical experience and relatively low costs [7]. There are various types of phytoconstituents present in the plants to treat this metabolic disorder. The plant of interest *Grewia hirsuta* (family: Tilaceae) has many phytochemical constituents such as Aldehyde, Alcoholic compound, α-Curcumene, Sesquiterpene, Sesquiterpene alcohol, Undecanoic acid,Tetradecanoic acid Myristic acid,Sesquiterpene oxide, n-hexadecanoic acid, Palmitic acid,Linoleic acid,Oleic Acid, Gingerol and Alkane which has been isolated from the leaf extract for the study of cardio protective potential [8]. The plant extract is used as anti-fertility [9], anti-ulcer and aphrodisiac agent [10]. Dried roots of this plant are administered along with few other ingredients to cure colic and rheumatic ailments in cattle [11]. For the first time, a compound (4*Z*, 12*Z*)-cyclopentadeca-4, 12-dienone with potent anti diabetic efficacy had been isolated from this plant that has been reported by the author in the earlier paper [12]. Molecular docking is an important computational tool to predict the plausible interactions between the drug and protein in a non-covalent fashion. Extensive *in silico* docking procedures have been carried out to examine whether the compound is a good ligand with diabetic targets such as Aldose reductase, Peroxisome proliferator-activated receptor-gamma, Glycogen synthase kinase-3, Pyruvate dehydrogenase kinase isoforms 2, Glucokinase, 11β-Hydroxysteroid dehydrogenase, Glutamine:fructose-6-phosphate amidotransferase.

Aldose reductase (ALR2; EC 1.1.1.21) (PDB ID 3G5E) is the rate-limiting enzyme in the Polyol pathway. It reduces excess D-glucose into D-sorbitol with the help of NADPH as a cofactor (El-Kabbani et al., 2004) [13]. It plays important role in diabetic microvascular complications (Kaul and Ramarao, 2001) [13,14]. Peroxisome proliferator-activated receptor-gamma (PDB ID 3DZY) key transcriptional factor plays a pivotal role in regulating adipogenesis, insulin sensitivity and glucose homeostasis [15,16]. Glycogen synthase kinase-3 (PDB ID 3F7Z) is a unique multifunctional serine/threonine kinase and it was inactivated by phosphorylation. In response to insulin binding, PKB/AKT phosphorylates GSK-3 on serine 9, which prevents the enzyme from phosphorylating glycogen synthase [17]. Unphosphorylated glycogen synthase is active and able to synthesize glycogen. Thus it plays a key role in the transduction of regulatory and proliferative signals arising out at the cell membrane in the insulin signalling pathway, leading to potential modulation of blood glucose levels [17]. Pyruvate dehydrogenase kinase isoforms (PDKs 1 - 4) (PDB ID 4MP2) negatively regulate activity of the mitochondrial pyruvate dehydrogenase complex (PDC) by reversible phosphorylation. PDK isoforms are up-regulated in obesity, diabetes, heart failure and cancer and are potential therapeutic targets for these important human diseases [18]. Glucokinase (hexokinase IV) has a major role in the control of blood glucose homeostasis because it is the predominant hexokinase expressed in the liver, has a very high control strength on hepatic glucose disposal, and is the glucose sensor for insulin secretion in pancreatic β-cells. Glucokinase (PDB ID-4IXC) is currently considered a strong candidate target for antihyperglycemic drugs for type 2 diabetes [19]. 11β-Hydroxysteroid dehydrogenase (11β-HSD) (PDB ID 4K1L) enzymes catalyze the conversion of biologically inactive 11-ketosteroids into their active 11β-hydroxy derivatives and vice versa. Inhibition of 11β-HSD1 has considerable therapeutic potential for glucocorticoid-associated diseases including obesity, diabetes, wound healing, and muscle atrophy [20,21]. Glutamine:fructose-6-phosphate amidotransferase (GFAT) (PDB ID 2ZJ4) is a rate-limiting enzyme in the hexoamine biosynthetic pathway and plays an important role in type 2 diabetes [22]. The enhanced activity of human GFAT has been implicated in insulin resistance in cellular and animal models. Thus, human GFAT is recognized as an interesting potential target for type 2 diabetes complications in medicinal chemistry [23]. To the best of our knowledge, this is the first report on docking studies of the compound (4Z, 12Z)-cyclopentadeca-4, 12-dienone isolated from *Grewia hirsuta* for antidiabetic activity.

## Methods

The fresh leaves of *Grewia hirsuta* plants were collected from Malachery forest, Gingee, Thiruvannamalai District, TamilNadu. The plant specimen was authenticated and voucher specimen (SRMU/BI/5) was deposited in the Herbarium at Proteomics lab, SRM University. The extraction was done from shade, dried and coarsely powdered leaves of the plant *Grewia hirsuta* (about 50gms) using methanol in a Soxhlet apparatus for 16 h. After column fractionation (E-Merck, Darmstadt, Germany and dimension of column was 450 x 30 mm) the compound (4*Z*, 12*Z*)-cyclopentadeca-4,12-dien-1-one with hypoglycemic activity was separated and was concentrated by a speed-vac under low pressure with evaporating temperature of 40°C. TLC was performed on a pre-coated silica gel TLC plates grade F254 (E-Merck, Darmstadt, Germany) to determine the number of compounds present in the given sample. The structure of the compound was elucidated and confirmed by spectroscopic methods including UV, FTIR (direct sampling method using TENSOR II FT-IR Spectrometer, Bruker, and Mumbai, India). ^1^H, ^13^C NMR (300 MHz) studies and the accurate mass determination were also carried out using the Q-TOF mass spectrometer available with electron spray ionization (ESI) (Micromass UK Ltd). All the above results were reported in the earlier paper by the author [12]. The elemental analysis was also carried out using Flash EA, Thermo Finnigan (Italy).

### Molecular docking analysis

The structures of the target receptor binding sites of human aldose reductase (PDB ID-3G5E), glucokinase, (PDB ID-4IXC), pyruvate dehydrogenase kinase (PDB ID-4MP2), peroxisome proliferator-activated receptor-gamma (PDB ID-3DZY), glycogen synthase kinase-3 (PDB ID-3F7Z),11β-Hydroxysteroid dehydrogenase (PDB ID-4K1L), glutamine:fructose-6-phosphate (PDB ID-2ZJ4) were retrieved from the RCSB Protein Data Bank (http://www.rcsb.org/pdb/home/home.do). The docking calculations were performed using the Schrödinger software suite with default settings if not indicated otherwise [24]. The proteins were prepared by using the Protein Preparation Wizard, pre-processed and heterostate for co-crystallized ligand was generated using Epik; protonation state and optimization of H bonding of the protein side chains were assigned using Protassign, energy minimization (impref minimization) was carried out using default constraint of 0.3 Å RMSD and OPLS 2005 force field. Receptor grid has been prepared with default parameters and without any constrains. The ligand was prepared by using Ligprep utility of Schrodinger Suite with default parameters, the ligand energy minimized by using OPLS 2005 (Macromodel multiple minimization) and water as solvent [24]. Ligand docking was performed using OPLS force field. In order to predict the binding affinity and pre eminent docked structures, the combined ligand docking and energy-grid scores were ranked by using E model and Glide scores. Docking was performed using the Extra Precision (XP) feature of GLIDE 5.0 module implemented in the Schrodinger LLC [24]. Visualization and analysis of protein–ligand complexes was performed using PyMOL ver. 0.99 software (De Lano Scientific LLC, CA, USA) [25]. The PDB Sum (http://www.ebi.ac.uk/pdbsum/) server was used to determine the active sites of the receptors and their interactions with the compound.

### ADME/T property analysis

The QikProp module of Schrodinger is a quick, accurate, easy-to-use absorption, distribution, metabolism, and excretion (ADME) prediction program design to produce certain descriptors related to ADME. It predicts both physicochemical significant descriptors and pharmacokinetically relevant properties. ADME properties determine drug-like activity of ligand molecules based on Lipinski’s rule of five. ADME/T properties of the compound (4*Z*, 12*Z*-Cyclopentadeca-4, 12-dien-1-one was analysed using Qikprop 3.2 module [26].

## Result and discussion

**Figure 1:**
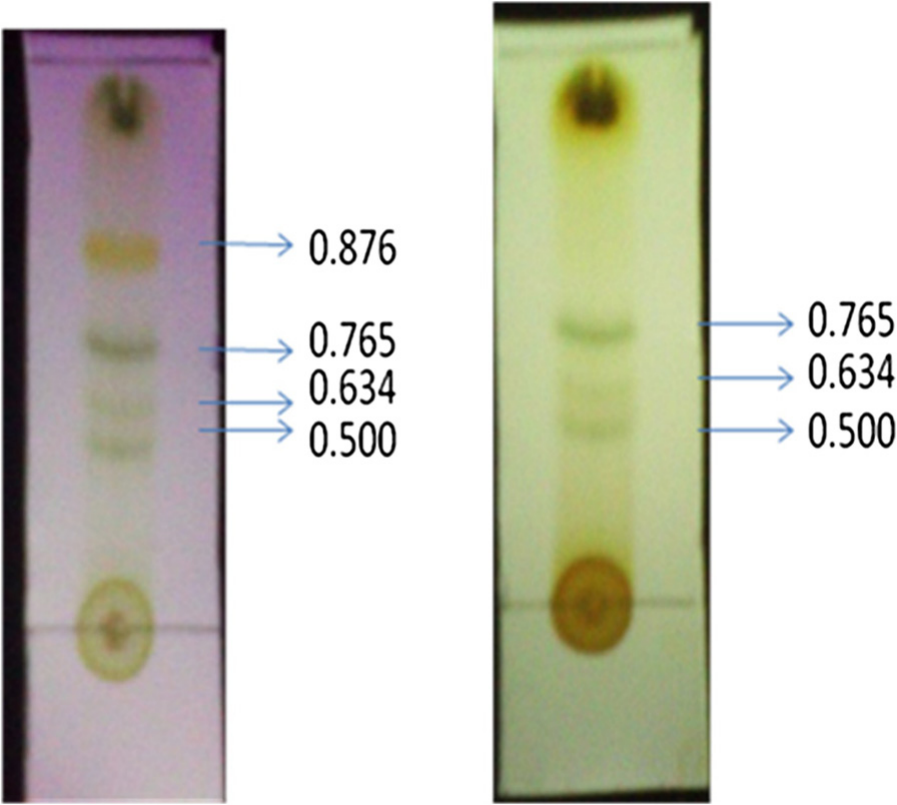
**Compounds separation by Thin layer chromatography.**

**Figure 2:**
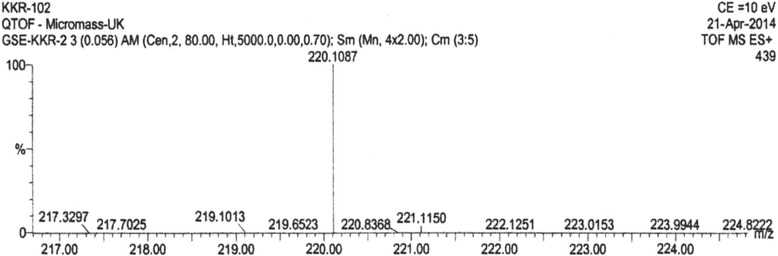
**Q-TOF mass spectrum of (4Z, 12Z)- Cyclopentadeca-4, 12-dienone.**

**Figure 3:**
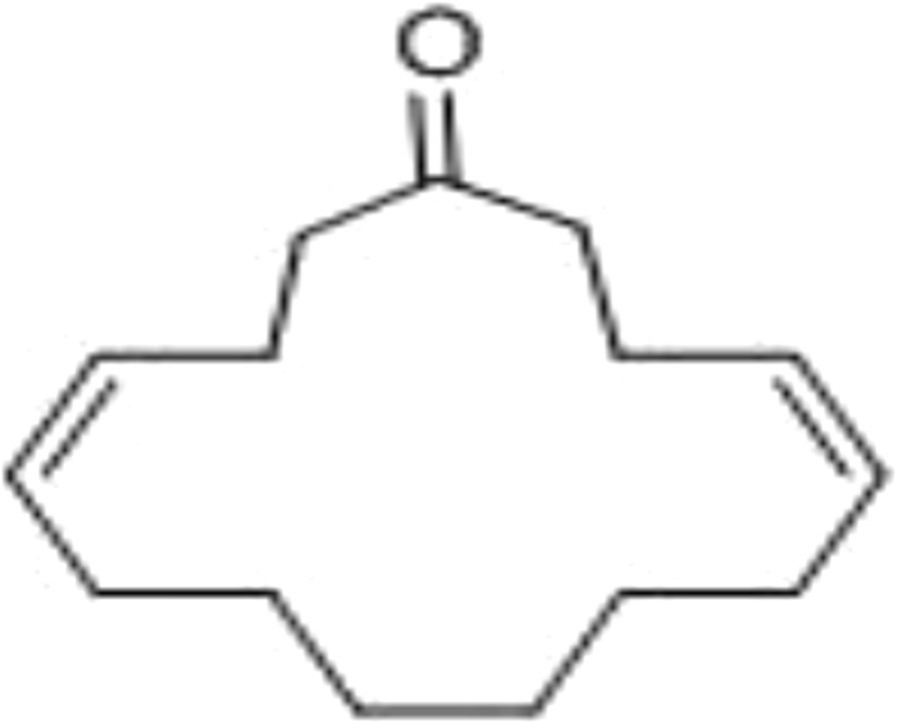
**Molecular structure of (4Z, 12Z)- Cyclopentadeca-4, 12-dienone.**

**Figure 4:**
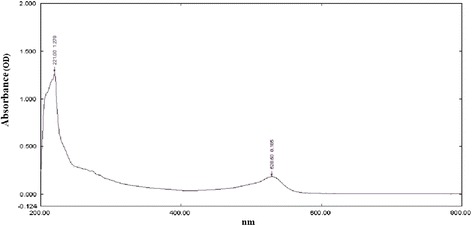
**UV-Vis spectrum of (4Z, 12Z)- Cyclopentadeca-4, 12-dienone.**

Under the influence of UV, four distinct compounds with R_*f*_ values 0.876, 0.765, 0.634 and 0.500 were recognized. When treated with iodine compounds with R_*f*_ values 0.765, 0.634 and 0.500 were seen. (4Z, 12Z)-cyclopentadeca-4, 12-dienone was an orange yellowish oily colored compound (yield: 650 mg) with the R_*f*_ value of 0.876 (9:1, chloroform: methanol) on TLC was observed with m.pt 68-69°C (Figure 1). Among the compounds, (4Z, 12Z)-cyclopentadeca-4, 12-dienone (R_*f*_ =0.876) gave good result of 52.3% α-amylase inhibitory activity and the other compounds gave only poor results on inhibitory effect [12]. The Q-TOF spectrum of (4Z, 12Z)-cyclopentadeca-4, 12-dienone showed a molecular ion peak at m/z 220 (100%,) (Figure 2) indicating the molecular weight with molecular formula is C_15_H_24_O (Figure 3). UV (methanol): λ_max_ = 221 nm (double bonds presence), at 528 nm (coloured compound) (Figure 4). FTIR studies: υ_max_ at 1711 cm^−1^ (presence of carbonyl group), 1641 cm^−1^ (–C = C), 2924–2853 cm ^−1^ (–CH _2_) [12]. The ^1^H NMR (CDCl_3_, 300 MHz) δ: 5.02-5.13 (m, olefinic protons), 0.86-2.10 (m, CH_2_ protons) The ^13^CNMR (CDCl_3_, 300 MHz) δ: 22.70- 39.77 ppm [presence of carbons in the aliphatic regions (-CH_2_)],173.77 (carbonyl carbon), 124.45 and 134.90 (olefinic carbons) [12]. The elemental analysis report revealed the presence of C (84.76%), H (8.98%) and O (6.26%) in the compound and absence of N.

**Figure 5:**
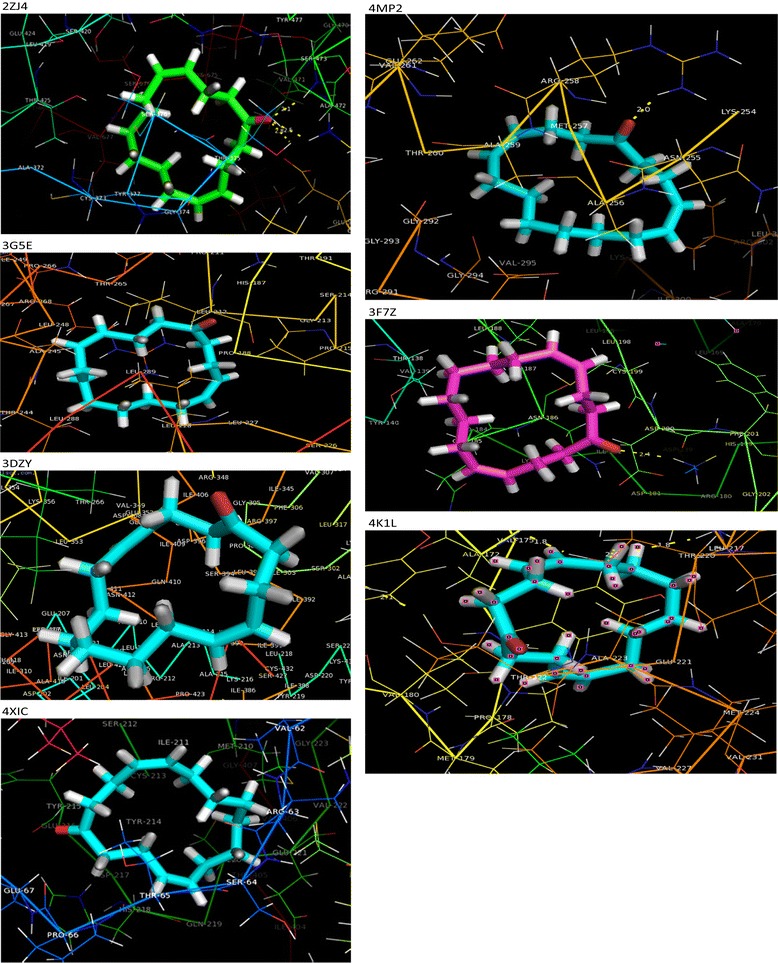
**Molecular docking of (4** ***Z*** **, 12** ***Z*** **)- Cyclopentadeca-4, 12-dien-1-one with the active site residues of selected target proteins.**

In order to investigate the binding capacity of bioactive compound in *Grewia hirsuta* on proteins related to diabetes in humans, we docked the compound to the target proteins. Results showed that the dock score ranged from -5.21 to -7.85 (kcal/mol) (Table 1). High binding affinity of the ligand to the receptor was explained clearly by interaction analysis (Figure 5). Glutamine: fructose-6-phosphate amidotransferase (GFAT) is the key enzyme in the hexoamine biosynthetic pathway and plays an important role in type 2 diabetes [22]. In our study, docking of novel compound with 2ZJ4 showed binding interaction of Keto group of the novel compound established the hydrogen bond with SER 473 which is the major interaction of the ligand with 2ZJ4. THR 425, SER 373, SER 422, SER 420, GLN 421 and SER 376 residues which are presented in the active site form strong polar interactions with the compound. ALA 472, LEU 556, GLY 374, VAL 471, VAL 677, CYS 373 form strong hydrophobic interactions. LYS 675, LYS 557 positively charged amino acids and negatively charged GLU 560 present in active site interacts with the compound. Similar interactions were observed in substrate 2-Deoxy 2- amino glucitol 6-phosphate interaction with GFAT [22].

**Table 1:** **Inhibitory activity of (4Z,12Z)-cyclopentadeca-4, 12-dienone on selected target proteins**

**S.No**	**Target protein**	**Ligand**	**Docking score**	**Glide emodel**	**Glide energy**
1	3G5E	(4Z,12Z)-cyclopentadeca-4, 12-dienone	−7.61	−42.27	−30.03
2	3DZY		−7.57	−43.74	−30.62
3	3F7Z		−6.01	−39.45	−28.72
4	4MP2		−5.21	−32.80	−26.26
5	4IXC		−6.18	−34.06	−22.81
6	4K1L		−7.85	−43.79	−26.06
7	2ZJ4		−5.57	−43.23	−32.54

The Molecular Docking analysis of the novel compound and the receptor 3DZY (intact PPAR gamma - RXR alpha Nuclear Receptor Complex on DNA bound with Rosiglitazone, 9-cis Retinoic Acid and NCOA2 Peptide) showed that ILE-268,CYS-432,PHE-439,LEU-694,VAL-342,VAL-265,ILE-345,VAL-349,PHE-346,PHE-313,ILE-310,ALA-272 residues form strong hydrophobic interactions and HIS 435 forms polar contact with the compound. Similar amino acid interactions are seen in potential retinoid X receptor targretin agonists for treating Alzheimer’s disease from traditional Chinese medicine [23].

Glucocorticoids (GCs) are potent functional antagonists of insulin action, and promote gluconeogenesis in the liver, potentially leading to raised blood glucose concentrations in diabetes. At the tissue level the access of active GCs to its receptors is governed by 11β-HSD1 [20]. The docking studies of 11β-HSD1with the compound makes reveals the best dock score of -7.85 kcal/mol and interact with hydrophobic residues such as ALA 226, Val 227, VAL 180, LEU 126, TYR 177, PRO 178, VAL 231, MET233 LEU 215, LEU 171, TYR 183 and ALA 223.GLY 216 and polar residue SER 170 are also involved in the interaction with the compound. These interactions clearly suggest that the compound fits very well into the binding pocket of 11β-HSD1. This conclusion is strongly supported by previous study on 1, 1-Dioxo-5, 6-dihydro-[1,2,4] oxathiazines, a novel class of 11ß-HSD1 inhibitors for the treatment of diabetes [21]. PDK plays a crucial role in glucose utilization and lipid metabolism through pyruvate dehydrogenase complex. In diabetes, PDK2 activity is markedly upregulated and PDK2 has been an emerging therapeutic target for type 2 diabetes as well as a candidate gene for that disease [18]. In our docking study of 4MP2 (Crystal structure of pyruvate dehydrogenase kinase isoform 2 in complex with inhibitor PA1) with the novel compound revealed that ARG 258 forms the strong hydrogen bond interaction with C = O of the novel compound. LEU 330,LEU 346,LEU 303, VAL 295,ALA 256, ALA 259 residues form hydrophobic interactions [27]. Polar residues ASN 255, THR 354and SER 263 present in the binding site interact with the compound. GLY at position 292 and 293 involved in interaction with the compound. Negatively charged ASP 290 and GLU 262 present in the binding pocket were found to be interacting with the compound. Our results coincide with the active site interaction of (5-Bromo-2, 4-Dihydroxyphenyl)(1,3-Dihydro-2 h-Isoindol- 2-Yl) methanone with ATP binding pocket [18,28].

Since aldose reductase inhibitors can play a significant role in preventing diabetic complications [29,30]. In the present study docking of 3G5E and the novel compound revealed PHE 122, PRO 218,TRP 219,LEU 300,CYS 298,TRP 111,TRP 48,TRP 79,VAL 47,TYR 48 residues form strong hydrophobic interactions. Polar residues GLN 49and HIS 110 present in active site also interact with the novel compound. The novel compound interacts in the similar manner like zenarestat inhibitor fits neatly in the hydrophobic active site and induces unique and dramatic conformational changes [31].

Glucokinase (GK) is expressed in various organs and plays a key role in hepatic glucose metabolism and pancreatic insulin secretion. GK could indeed serve as pacemaker of glycolysis and could be an attractive target for type 2 diabetes (T2D) [17]. In the present study the novel compound reveals strong hydrophobic interaction with residues VAL 62, CYS 252,ILE 211,VAL 452,VAL 455,PRO 66,THR 214,MET 235 and MET 210 . Positively charged ARG 63, negatively charged GLU 67 and GLU 227 interact with novel compound respectively. Polar residues present in the binding pocket SER 64, THR 65 interacts with the novel compound. Similar interactions were observed in (2 s)-2-{[1-(3-Chloropyridin-2-Yl)-1 h-Pyrazolo [3,4-D]pyrimidin-4-Yl] oxy}-N-(5-Methylpyridin-2-Yl)-3-(Propan-2-Yloxy) propanamide inhibitor with 4IXC [32]. Our docking results. The binding interactions and involvement of specific amino acid could lead to design of novel glucokinase activator with higher selectivity and least side effects for the treatment of type 2 diabetes and related disorders.

GSK-3 plays a key role in the regulation of beta cell mass and is a target for beta cell regenerative therapies. Docking study on Glycogen Synthase Kinase 3beta (PDB Code 3F7Z) and the novel compound revealed that the oxygen atom of the novel compound reacts with LYS-85 and forms hydrogen bond. Strong hydrophobic interaction was observed around the cyclopentane ring such as near the VAL-110,LEU-132,ALA-83,LEU-188,VAL-135,CYS-199 and PHE-67, polar interactions also occurs with amino acids such as Thr 138 and Asn-186 [33].

### ADMET predictions

The drug-like activity of the ligand molecule is characterized using ADME properties. ADME prediction can be used to focus lead optimization efforts to enhance the desired properties of a given compound. The expected ADME property of the tested compound was evaluated with QikProp module of Schrodinger (Table 2). The selected properties are known to influence metabolism, cell permeation, and bioavailability. Almost all the predicted properties of the tested compound was in the range as predicted by QikProp for 95% of known oral drugs and also satisfy the Lipinski’s rule of five to be considered as drug like potential.

**Table 2:** **Qikprop admet property results**

**Entry name**	**Human oral absorption** ^**a**^	**QPlogPo/w** ^**b**^	**CNS** ^**c**^	**mol MW** ^**d**^	**donorHB** ^**e**^	**accptHB** ^**f**^	**Rule of five** ^**g**^
(4Z, 12Z)- Cyclopentadeca-4, 12-dienone	80	4.393	−2	220	2	4	0

^a^% Human oral absorption: <25% is poor absorption. ^b^QPlogPo/w: Partition coefficient; recommended range -2.0 - 6.5. ^c^CNS: Predictive Central Nervous Activity -2 (inactive) to +2 (active). ^d^M.wt.: Molecular weight of the molecule < 500 is preferred. ^e^donor HB: Estimated number of hydrogen bonds that would be donated by the solute to water molecules in an aqueous solution, recommended range 0.0 - 6.0 ^f^accpt HB: Estimated number of hydrogen bonds that would be accepted by the solute from water molecules in an aqueous solution, recommended range 2 - 20. ^g^Rule of five: Number of violations of Lipinski’s rule of five; recommended range 0 - 4.

## Conclusion

Docking studies of the (4Z, 12Z)- Cyclopentadeca-4, 12-dienone with seven different target proteins showed that this is a promising candidate which docks well with various targets related to diabetes mellitus. ADME properties can be taken as best hit molecule and can be considered for further studies like QSAR. Thus (4Z, 12Z) - Cyclopentadeca-4, 12-dienone can be considered for developing into a potent antidiabetic drug.
